# Supplementary material for: Baseline Characterization of the Gut Microbiota of Field and Colony Populations of Phlebotomus tobbi and Preliminary Assessment of the Anti-Leishmanial Activity of Cultivable Bacteria
Source: Pathogens. 2026 Jun 23;15(7):658. doi: 10.3390/pathogens15070658 (PMC13416292; doi:10.3390/pathogens15070658)
Supplement: Supplementary file 1 [file pathogens-15-00658-s001.zip › pathogens-4357168-File S1/Composition_Barplots/Figures/charts/i69PNphbJBp8OrOnF6UKXZ4Fn1MDr9_legend.pdf]

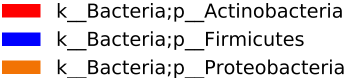
k__Bacteria;p__Actinobacteria
k__Bacteria;p__Firmicutes
k__Bacteria;p__Proteobacteria
